# Factors Affecting Microcuttings of *Stevia* Using a Mist-Chamber Propagation Box

**DOI:** 10.1155/2013/940201

**Published:** 2013-12-29

**Authors:** Mohamad Osman, Nur Syamimi Samsudin, Golam Faruq, Arash Nezhadahmadi

**Affiliations:** ^1^Faculty of Plantation and Agrotechnology, Universiti Teknologi MARA, 40450 Shah Alam, Selangor, Malaysia; ^2^Faculty of Science, International Islamic University, 25200 Kuantan, Pahang, Malaysia; ^3^Institute of Biological Sciences, Faculty of Science, University Malaya, 50603 Kuala Lumpur, Malaysia

## Abstract

*Stevia rebaudiana* Bertoni is a member of Compositae family. *Stevia* plant has zero calorie content and its leaves are estimated to be 300 times sweeter than sugar. This plant is believed to be the most ideal substitute for sugar and important to assist in medicinal value especially for diabetic patients. In this study, microcutting techniques using a mist-chamber propagation box were used as it was beneficial for propagation of *Stevia* and gave genetic uniformity to the plant. The effects of different treatments on root stimulation of *Stevia* in microcuttings technique were evaluated. Treatments studied were different sizes of shoot cuttings, plant growth regulators, lights, and shades. Data logger was used to record the mean value of humidity (>90% RH), light intensity (673–2045 lx), and temperature (28.6–30.1°C) inside the mist-chamber propagation box. From analysis of variance, there were significant differences between varieties and treatments in parameters studied (*P* < 0.05). For the size of shoot cuttings treatment, 6 nodes cuttings were observed to increase root number. As compared to control, shoot cuttings treated with indole butyric acid (IBA) had better performance regarding root length. Yellow light and 50% shade treatments showed higher root and leaf number and these conditions can be considered as crucial for potential propagation of *Stevia*.

## 1. Introduction

*Stevia*, botanically known as *Stevia rebaudiana* Bertoni, is a small perennial herb with mid green leaves. It is derived from the tropics and subtropical South America and Central America. *Stevia* is cultivated in several countries such as Brazil, Canada, China, Japan, Paraguay, and South East Asia. It is a natural sweetener plant and the leaves are estimated to be 300 times sweeter than sugar (sucrose). The worldwide demand for high potency sweeteners, particularly natural sweeteners is expected to increase year by year [1]. *Stevia* is not only a natural calorie free plant but also has other advantages as chemical artificial sweeteners, such as aspartame, cyclamate, and saccharine. The leaves produce diterpene glycosides (stevioside and rebaudiosides) which are nonnutritive, nontoxic, and high-potency sweeteners and may substitute sucrose as well as other synthetic sweeteners. The sweetness is due to stevioside which is the most abundant glycoside [2]. *Stevia* has also medicinal values in many therapeutic applications such as dental health, diabetes, hypoglycemic, indigestion, obesity, yeast infection, oral health, skin toning, and healing burns and wounds [3]. Vegetative propagation methods like air layering, budding, and cutting are being widely applied to maintain their purity for commercial exploitation and multiply plants of desired genetic constitution. However, in micropropagation, rooting of cuttings is often problematic. There are many species which are difficult to root and many genotypes are recalcitrant to root in conventional propagation via cuttings [4]. Microcutting technique using mist-chamber propagation box was employed in mist-chamber propagation box in order to evaluate the rooting formation of *Stevia*'s cuttings. According to Awang et al. [5], roots can be either initiated from the stem or at the based-stem and the number of adventitious roots produced by microcutting was significantly different for different species. In addition, the use of microcutting for propagation of different plant species has been a popular technique and equally effective. In this technique, plant material used comprised of stem cutting obtained from juvenile mother plants raised from micropropagation grown in high planting density under partially protected condition and pruned regularly. However, this microcutting technique still needs exploration and improvement in order to increase rooting performance in plants. As the seeds of *Stevia* are very small in size and infertile, large scale mechanized production of *Stevia* through seeds is not fruitful [6]. The poor seed germination problem in this crop made a lot of obstacles towards large scale establishment of the crop and thereby making the available plant materials costly. Therefore, it is the best to propagate *Stevia* cuttings from a plant that has proven to be successful [7]. Cutting propagation is simply the excision of plant part (stem, root, or leaf cuttings) and nurturing the part to grow into a genetic replica of the original or parent plant [8]. In addition, cutting enables researchers to clone selected genotypes and test the effects of various treatments among genetically identical individual. Thus, it can reduce the variation in intrinsic properties among individual plants [9]. Microcutting sizes of specific plant are believed to be one of the factors that could affect rooting performance of such plants. According to Haq et al. [10], different microcutting sizes like uninodal (1 node), binodal (2 nodes), trinodal (3 nodes), and tetranodal (4 nodes) of olive plants gave different rooting and shooting formations. Rooting can be enhanced by using plant growth regulators and auxins have been used for root initiation in cuttings of various horticultural crops. Auxins play a critical role in plant cell growth and are involved in a wide variety of developmental processes including initiation of apical dominance, fruit development, lateral root production, leaf primordial, and phototropism. Among them, indole butyric acid (IBA) and naphthalene acetic acid (NAA) are widely used. In *Stevia*, auxin treatment is one of the factors that affects rooting of shoots as it is known to be involved in rooting over a long period of time [10]. IBA is used in many crops and ornamentals to promote growth and development of flowers, fruits, and roots. Haq et al. [10] stated that IBA is mostly used to stimulate rooting in microcutting due to its weak toxicity and great stability. Another research by Ingle [2] also mentioned that treating *Stevia* with IBA promoted rooting and increased the root number. NAA has been determined to be highly effective in initiating root formation. It significantly increases the number, length, and dry weight of root hairs. It also induces early flowering and improves cell splitting and expansion [11]. Previous study by LaPierre [9] showed that NAA had effect on adventitious root production in cuttings of *C. obtusifolia*. Light can be measured on the light scale that ranges from red to blue. Although all plants need light in the blue spectrum to grow, the amount of light required depends on the classification of the plant. The energy contained in light is absorbed in the chlorophyll of plants. Color affects plant growth and light modifies plants development through various signal transduction networks [12]. Plants rely on lights in order to go through the process of photosynthesis which allows plants to create their own food. Phytochrome pigments absorb red and far red portions of the light spectrum. Therefore, they can regulate dormancy, root development, seed germination, tuber and bulb formation, flowering, and fruit production. Moreover, red light has been shown to enhance leaf carbohydrate concentrations [12]. Yellow light is a mixture of green and red light. Plants reflect green and yellow lights which are in the middle of the color spectrum so that these two wavelengths have little effect on plant growth. Since yellow is weakly absorbed by the pigments, plants may respond slowly. According to Guerra [13], yellow light in a plant is not enough to allow a plant to make enough energy to thrive. However, little information on the effect of white and yellow lights towards the rooting performance in *Stevia* is provided previously. *Stevia* is a sun loving plant since the plant thrived in humid, warm, and sunny climate. Under natural habitat, it grows wild along with tall grasses under partial shade. However, planting of *Stevia* cuttings in the field under direct sunlight has limited success [2]. Therefore, there is still lack of review about the effect of shade on rooting performance in plants. Due to the increasing demand of the world, effective propagation technique for *Stevia* is required for maintaining genetic stability and propagation of the same clones. In this study, the influence of different treatments on microcutting of *Stevia* (different size of shoot cuttings, plant growth regulators, lights, and shades) was evaluated. Furthermore, root and shoot developments from microcutting of *Stevia* together with appropriate factors that promote microcutting of *Stevia* were identified.

## 2. Materials and Methods

### 2.1. Plant Materials

The mother plants of three *Stevia* varieties including Mergong, MS007, and MS012 were obtained from Faculty of Science, International Islamic University, Malaysia. For each variety, shoot cuttings were made in a slant form and propagated using mist-chamber propagation box for root stimulation. The cuttings that formed roots were transferred into the poly bag which contained clay, sand, and organic matter in 1 : 1 : 1 ratio.

### 2.2. Preparation of Mist-Chamber Propagation Box

The mist-chamber propagation box was designed according to Awang et al. [5]. This chamber box was constructed using polystyrene boxes (55 cm × 43 cm × 30 cm) and covered with polyethylene sheet to maintain a humid environment (>90%). The box contained a medium of perlite at upper level and light expanded clay aggregates (LECA) at the base level. A water reservoir was created and maintained at the bottom of the box and water would then flow through capillary rise into perlite. A hole was thumbed on sidewall at 5 cm above the base of each box so that a good air-water relationship atmosphere existed in the rooting zone. This box was filled with water up to designated level before the insertion of shoot cuttings. Then, the box was covered by designed lid that could allow light penetration into the box. Humidity, light intensity, and temperature inside the mist-chamber propagation box were recorded by using data logger.

### 2.3. Application of Treatments

Shoot cuttings of *Stevia* were harvested using a microcutting technique from mother plants after one month of establishment. The cuttings were then inserted into mist-chamber propagation box and left for some days while observations continued. Insertion of treated cutting was done in such a way that the distal node was buried in the perlite layer. Shoot cuttings from three varieties of *Stevia* were treated with 4 treatments such as different sizes of shoot cuttings, plant hormones, and light and shade percentages. 2, 4, and 6 nodes of *Stevia* shoot cuttings from three different varieties were used in which each of the nodes had five replications for each variety and was propagated using three different mist-chamber propagation boxes. The cuttings were directly inserted into perlite in the box for rooting stimulation. Shoot cuttings in two different mist-chamber propagation boxes were treated with plant growth regulators. The excised cuttings were dipped into IBA and NAA powder for two seconds and allowed to stay for two minutes in order for the auxin to be absorbed into the cut shoot through the xylem. These treated shoot cuttings were then inserted into the mist-chamber propagation boxes in order to sprout roots. The shoot cuttings in other mist-chamber propagation boxes served as control experiment and were directly inserted into perlite without being treated with any plant growth regulators. For the light treatment, cuttings in three different mist-chamber propagation boxes were placed under red, white, and yellow lights. The shoot cuttings in other mist-chamber propagation box represented a control experiment and placed under the sunlight (normal condition). Finally, for shade treatment, the shoot cuttings in two mist-chamber propagation boxes were directly inserted into perlite and then covered by 50% and 100% of black net on the top of boxes. The function of different percentages of black nets was to give limited amount of sunlight penetration to the plant in the boxes. The shoot cuttings in other mist-chamber propagation box represented a control experiment and not covered by a black net.

### 2.4. Data Collection

Roots development for each cutting was recorded within 14 days. The data was collected 5 times within this period after 6 days of the experiment initiation. Roots growth was monitored by counting the root number produced after insertion of *Stevia*'s shoot cuttings into the rooting medium. Rooted cuttings were recorded and the shoot cuttings were then carefully returned back into the medium and noted after finishing the counting. At the same time, leaf number generated by shoots was counted and recorded from the beginning to the end of the experiment.

### 2.5. Statistical Analysis

Minitab 15 and SPSS 16.0 software were used for statistical analysis.

## 3. Results and Discussion

### 3.1. The Effect of Different Sizes of Shoot Cuttings

Root number, root length, and leaf number among varieties and node cuttings treatment from day 6 until day 14 in mist-chamber propagation box are presented in Tables 1, 2, and 3, respectively. Based on the results obtained, higher root number, root length, and leaf number were observed on day 14 compared to other days. Therefore, only data from day 14 was subjected to analysis of variance in order to determine significant differences between treatments (Table 4).

#### 3.1.1. Root Number

It was revealed that root number produced by microcutting was significantly different between varieties and nodes cuttings (*P* < 0.05) (Table 4). From Table 1, all varieties showed different root numbers in which MS012 variety showed greater root number compared to Mergong and MS007. For microcutting sizes, 2 node cuttings gave lower root number compared to 4 and 6 nodes cuttings and the mean of 6 nodes cuttings was higher than 4 nodes cuttings. However, the varieties and nodes did not interact with each other in terms of roots number (Table 4). The results obtained were supported by Haq et al. [10] who stated that the cuttings with more number of nodes would increase root number. Supposedly, this outcome might be due to the sufficient carbohydrate reserved in plants as 2 nodes cuttings produced less root number due to the lack of carbohydrate. Carbohydrate is important for root initiation which is energy requiring process [2].

#### 3.1.2. Root Length

There were significant differences between varieties and nodes cuttings in root length (*P* < 0.05) (Table 4). It was observed that MS007 showed the lowest root length compared to Mergong and MS012 (Table 2). However, MS012 developed greater root elongation than Mergong. The cuttings from 6 nodes had higher root length followed by 4 and 2 nodes cuttings, respectively. Higher root length might be due to the ability of their cells to undergo more differentiations than other nodes cuttings. In addition, division of cell also leads to the root elongation. Moreover, energy reserved in 2 and 4 nodes cuttings might be utilized only for root initiation and remained insufficient for elongation process; hence they produced shorter roots.

#### 3.1.3. Leaf Number

Leaf number was significantly different between varieties and nodes (*P* < 0.05) (Table 4). All varieties including Mergong, MS007, and MS012 had different leaf numbers (Table 3). MS007 possessed more leaf numbers compared to other varieties and leaf number was observed to be higher in 6 nodes cuttings compared to 2 and 4 nodes cuttings. Meanwhile, 2 nodes cuttings showed the lowest leaf number. However, there was no interaction between varieties and nodes regarding leaf number. This might be due to the activation of shoot growth which probably increased leaf number. Therefore, 6 nodes cuttings were preferable for *Stevia* to produce more leaves in microcutting technique.

### 3.2. The Effect of Plant Growth Regulators on Shoot Cuttings

Tables 5, 6, and 7 show root number, root length, and leaf number among varieties and plant growth regulators from day 6 to day 14 in mist-chamber propagation box. From these tables, all parameters studied from day 14 showed higher responses compared to the data from other days. Therefore, only data from day 14 was subjected to analysis of variance in order to determine significant difference between treatments (Table 8).

#### 3.2.1. Root Number

According to the results obtained, there were significant differences between varieties and plant growth regulators in root number (*P* < 0.05) (Table 8). MS012 produced more root number compared to Mergong and MS007 (Table 5). Besides, the highest root number was observed in the shoot cuttings treated with IBA followed by NAA and control, respectively, in all varieties. It was also observed that varieties and plant growth regulators did not interact with each other. It can be convinced that IBA was suitable for root formation in *Stevia* in comparison with NAA. The results were also supported by Ingle and Venugopal [14] who mentioned that treating *Stevia*'s cutting with IBA promoted root number. IBA was able to enhance tissue sensitivity and increased root number. Moreover, increasing root number might be due to enhanced hydrolysis of carbohydrates caused by auxin treatment.

#### 3.2.2. Root Length

There were significant differences between varieties and plant growth regulators in root length (*P* < 0.05) (Table 8). Mergong variety had the lowest root length compared to MS007 and MS012 (Table 6). The root length was found to be superior in all varieties shoot cuttings treated with IBA followed by NAA and control. However, the results showed that there was no correlation between varieties and plant hormones with each other. Higher root length in shoot cuttings were observed when treated with IBA and it might be occurred due to the cell elongation and division in suitable growing condition. Activity of auxin in *Stevia* plant might have caused hydrolysis and translocation of nitrogenous substances at the base of cutting [2]. Another possible reason might be because of the early formation of roots. The results obtained in this study were the same as the study conducted by Jadhav [15] on *Grewia subinaequalis* in which the cuttings treated with IBA produced the longest roots.

#### 3.2.3. Leaf Number

Table 8 shows that there were no significant differences between varieties and plant regulator hormones in leaf number. The results also showed no correlation between both treatments with each other. These indications showed that leaf number was not affected by varieties and plant hormones.

### 3.3. The Effect of Lights on Shoot Cuttings

Root number, root length, and leaf number among varieties and light treatments from day 6 to day 14 in mist-chamber propagation box are presented in Tables 9, 10, and 11, respectively. Higher root number, root length, and leaf number were observed on day 14 compared to other days. Therefore, only results from day 14 were subjected to analysis of variance in order to determine significant differences between treatments (Table 12).

#### 3.3.1. Root Number

**Figure 1:**
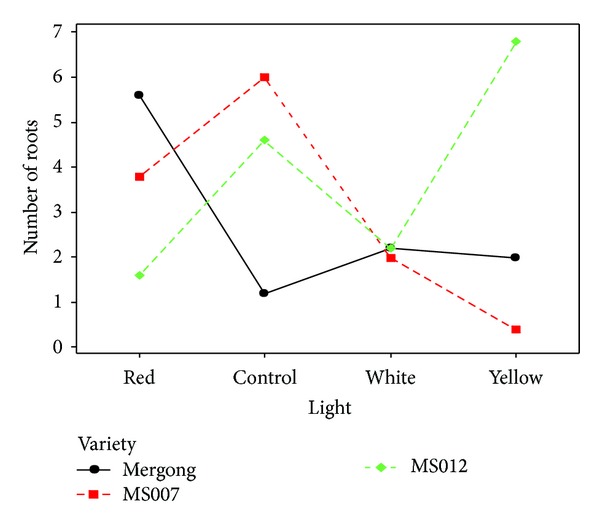
Interactions between varieties and light treatments in root number on day 14.

It was revealed that interactions between varieties and lights were significantly different in the root number (*P* < 0.05) (Table 12). The results obtained showed that different varieties and lights had different effects on root number. This was because different wavelength of lights could influence plants' growth in different ways. Based on Figure 1, almost similar responses were observed in all varieties when they are exposed to white light treatment. MS007 had a decreased root number compared to control in all light treatments. Instead, only shoot cuttings from Mergong were suitable to be exposed to all light treatments as it had better root number compared to the control. However, higher root number was observed in MS012 when exposed to yellow light. In general, red light had better root number in most varieties in this study. This was due to the phytochrome which monitored red light to have strong effect on root growth. However, not all varieties of *Stevia* except for Mergong and MS012 can be exposed to red and yellow lights, respectively, to produce more roots.

#### 3.3.2. Root Length

**Figure 2:**
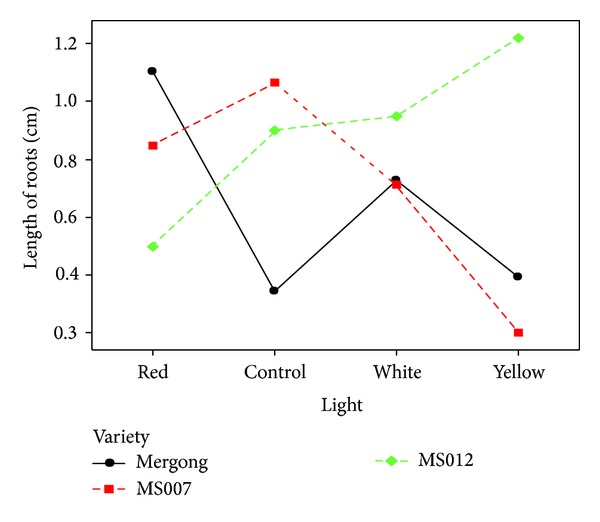
Interactions between varieties and light treatments in root length on day 14.

It was revealed that root length was significantly different among varieties and light treatments (*P* < 0.05) (Table 12). Based on Figure 2, MS007 had greater root length in control compared to red, white, and yellow lights, respectively. In contrast, Mergong performed better root length in all light treatments in comparison with control. MS012, when exposed to white and yellow lights, gave higher root length than control and red light accordingly. The longer roots produced might be due to better cell division and differentiation.

#### 3.3.3. Leaf Number

There was no significant difference between varieties and lights in leaf number (Table 12). The result obtained also showed no interactions between treatments. These indications demonstrated that varieties and lights did not affect emergence of new leaves.

### 3.4. The Effect of Shades on Shoot Cuttings

Tables 13, 14, and 15 show root number, root length, and leaf number, respectively, among varieties and light treatments from day 6 to day 14 in mist-chamber propagation box. Results from day 14 showed higher response in terms of root number, root length, and leaf number as compared to other days. Therefore, only results from day 14 were subjected to analysis of variance in order to determine significant differences between treatments (Table 16).

#### 3.4.1. Root Number

**Figure 3:**
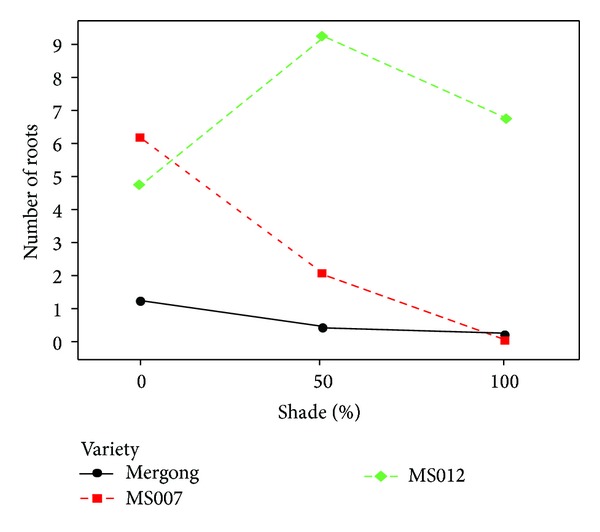
Interactions between varieties and shade treatments in root number on day 14.

It was revealed that there were significant differences in terms of root number in interaction of varieties and shade treatments (*P* < 0.05) (Table 16). From Figure 3, Mergong and MS007 when treated with 50% and 100% shade had lower root number compared to the control. However, 50% and 100% shade treatments increased root number in MS012. Light is necessary for plants during photosynthesis process. It helps produce foods in the form of carbohydrate which is used for plant growth and structural development. Root initiation in *Stevia* can also occur in darkness; hence this plant increases root number. The results were supported by Ingle [2] who claimed that direct planting of *Stevia* cuttings in the field under sunlight can limit success. However, decreasing root number in Mergong and MS007 when exposed to shade treatments revealed that these varieties could not undergo root initiation process. Photosynthesis might be stopped due to the little amount penetration of the sunlight into the shoot cuttings inside the mist-chamber propagation box.

#### 3.4.2. Root Length

There were significant differences between varieties and shade treatments in root length (*P* < 0.05) (Table 16). Higher root length was only observed in MS012 when treated with 50% shade compared to the control. So, MS012 was reliable for 50% shade treatment as it gave greater root elongation. Root elongation might be due to sufficient utilization of nutrients by shoot cuttings in mist-chamber propagation box for development of roots [16].

#### 3.4.3. Leaf Number

There was no significant difference among varieties in leaf number (Table 16). The effects of varieties and shades on leaf number are shown in Table 15. However, it was revealed that there was a significant difference among different shade treatments in leaf number (*P* < 0.05) (Table 16). According to Table 15, in all varieties, leaf number was decreased in 100% shade treatment compared to control and 50% shade treatments. Higher leaf number was only observed in 50% shade treatment. However, 50% shade treatment can be suitable to assist *Stevia*'s growth in microcutting technique. Moreover, increasing too much shade was not good as it would reduce photosynthesis rate production of new leaves.

## 4. Conclusion

Microcutting technique is believed to be a reliable method to increase rooting stimulation of *Stevia*'s shoot cuttings in mist-chamber propagation box. All of the treatments in this research were contributed to the development of shoot cuttings of *Stevia*. Based on results obtained, there were significant differences between varieties and treatments in parameters studied. In the current investigation, 6 nodes cuttings are preferred to be used in microcutting technique as they showed greater root number in the size of shoot cuttings treatment. The good responses in the parameters studied suggested that IBA treatment was reliable to induce root formation in *Stevia* plant. Moreover, growth performance of *Stevia*'s shoot cuttings was also improved by application of different light treatments. Besides, 50% of shade treatment was suitable for root stimulation of *Stevia*'s shoots cuttings. So, the results achieved in this study can be useful for upcoming research projects especially in other *Stevia* genotypes. In order to achieve mass production of *Stevia* and facilitate commercialization in economic rate, utilization of microcutting technique is the best method to be employed. This is because propagation of *Stevia* through this technique allows researchers to clone selected genotypes. Several improvements can be made to improve root stimulation of *Stevia*'s shoot cuttings. For example, the influence of combination of all treatments should be tried to get better results. Furthermore, to get more reliable outcomes, data collections should be extended for other varieties of *Stevia*. Last but not least, combination of IBA and NAA treatments with different concentrations can be applied in order to see proper growth of shoot cuttings.

## Figures and Tables

**Table 1:** Mean root number of three varieties having 2, 4, and 6 nodes cuttings from day 6 to day 14.

Variety	Nodes	Root number				
		Day 6	Day 8	Day 10	Day 12	Day 14
Mergong	2	3.20 ± 1.02	4.60 ± 0.68	4.60 ± 0.68	4.80 ± 0.66	5.00 ± 0.84^e^
	4	3.60 ± 0.51	4.80 ± 0.59	6.20 ± 0.97	6.40 ± 0.93	6.60 ± 1.03^cd^
	6	3.40 ± 0.51	5.80 ± 0.74	6.40 ± 0.60	6.40 ± 0.60	6.60 ± 0.68^cd^

MS007	2	2.80 ± 0.38	3.00 ± 0.32	4.00 ± 0.71	5.20 ± 1.16	6.00 ± 1.10^d^
	4	5.20 ± 0.86	5.80 ± 0.74	6.20 ± 0.86	6.80 ± 0.74	7.60 ± 0.93^b^
	6	5.00 ± 0.63	6.60 ± 0.40	7.00 ± 0.37	8.20 ± 0.49	9.40 ± 0.81^a^

MS012	2	6.20 ± 0.74	6.20 ± 0.74	6.60 ± 0.75	6.60 ± 0.75	6.80 ± 0.80^c^
	4	5.40 ± 0.51	7.20 ± 0.58	8.40 ± 0.75	8.60 ± 0.87	9.00 ± 0.89^ab^
	6	7.40 ± 0.87	8.00 ± 1.14	8.00 ± 1.14	8.40 ± 1.12	9.60 ± 0.68^a^

*Note*. Mean value within a column followed by the same letter shows no significant difference at *P* < 0.05 using Tukey test.

**Table 2:** Mean root length of three varieties having 2, 4, and 6 nodes cuttings from day 6 to day 14.

Variety	Nodes	Root length (cm)				
		Day 6	Day 8	Day 10	Day 12	Day 14
Mergong	2	0.60 ± 0.09	1.05 ± 0.11	1.34 ± 0.12	1.50 ± 0.14	1.85 ± 0.16^bc^
	4	0.54 ± 0.06	1.04 ± 0.11	1.35 ± 0.14	1.67 ± 0.14	1.95 ± 0.18^b^
	6	0.49 ± 0.08	0.97 ± 0.08	1.54 ± 0.10	1.93 ± 0.12	2.58 ± 0.17^a^

MS007	2	0.35 ± 0.04	0.57 ± 0.05	0.60 ± 0.07	0.78 ± 0.07	0.83 ± 0.07^d^
	4	0.39 ± 0.03	0.56 ± 0.05	0.66 ± 0.08	0.84 ± 0.08	0.94 ± 0.09^cd^
	6	0.42 ± 0.03	0.42 ± 0.03	0.68 ± 0.06	0.90 ± 0.09	1.20 ± 0.07^c^

MS012	2	0.63 ± 0.04	1.07 ± 0.07	1.33 ± 0.10	1.81 ± 0.14	2.02 ± 0.16^b^
	4	0.85 ± 0.08	1.17 ± 0.08	1.56 ± 0.11	2.01 ± 0.14	2.28 ± 0.14^ab^
	6	1.20 ± 0.07	1.66 ± 0.09	2.00 ± 0.12	2.57 ± 0.13	2.76 ± 0.12^a^

*Note*. Mean value within a column followed by the same letter shows no significant difference at *P* < 0.05 using Tukey test.

**Table 3:** Mean leaf number of three varieties having 2, 4, and 6 nodes cuttings from day 6 to day 14.

Variety	Nodes	Leaf number				
		Day 6	Day 8	Day 10	Day 12	Day 14
Mergong	2	1.00 ± 0.63	4.00 ± 0.63	4.40 ± 0.40	4.40 ± 0.40	5.20 ± 1.20^d^
	4	2.40 ± 0.75	4.40 ± 0.98	5.60 ± 0.81	6.00 ± 0.51	6.00 ± 0.58^c^
	6	2.00 ± 0.00	4.00 ± 0.45	4.60 ± 1.03	6.40 ± 0.75	8.00 ± 1.10^bc^

MS007	2	2.80 ± 0.49	4.00 ± 0.00	5.40 ± 0.40	5.80 ± 0.49	5.80 ± 0.66^cd^
	4	2.80 ± 0.49	4.20 ± 0.37	5.20 ± 0.37	6.60 ± 0.40	8.20 ± 0.37^b^
	6	3.20 ± 0.63	6.00 ± 0.75	8.80 ± 0.97	10.40 ± 1.20	12.20 ± 1.24^a^

MS012	2	0.00 ± 0.00	0.20 ± 0.20	0.20 ± 0.20	0.20 ± 0.20	1.00 ± 0.63^f^
	4	0.00 ± 0.00	1.60 ± 0.98	2.40 ± 0.98	2.40 ± 0.98	3.80 ± 1.20^e^
	6	1.60 ± 0.75	3.00 ± 0.55	3.60 ± 0.68	4.80 ± 0.80	5.80 ± 0.74^cd^

*Note*. Mean value within a column followed by the same letter shows no significant difference at *P* < 0.05 using Tukey test.

**Table 4:** Mean square from analysis of variance for root number, root length, and leaf number on day 14.

Source	df	Root number	Root length	Leaf number
Variety	2	22.40**	8.27**	82.76**
Nodes	2	26.60**	1.76**	56.16**
Variety∗nodes	4	1.50^ns^	0.07^ns^	1.89^ns^
Error	36	3.80	0.15	4.16

Total	44			

*Note*. *indicates significant difference at *P* < 0.05; **indicates significant difference at *P* < 0.01; ^ns^indicates no significant difference at *P* > 0.05.

**Table 5:** Mean root number of three varieties treated with IBA and NAA from day 6 until day 14 in mist-chamber propagation box.

Variety	Plant hormone	Root number				
		Day 6	Day 8	Day 10	Day 12	Day 14
Mergong	Control	0.00 ± 0.00	0.40 ± 0.40	0.40 ± 0.40	0.40 ± 0.40	1.20 ± 0.58^gh^
	IBA	2.40 ± 2.40	3.00 ± 3.00	3.60 ± 3.60	4.00 ± 4.00	5.60 ± 4.86^e^
	NAA	0.00 ± 0.00	0.00 ± 0.00	0.60 ± 0.40	1.20 ± 0.97	1.80 ± 1.36^g^

MS007	Control	0.80 ± 0.58	2.60 ± 1.08	4.00 ± 1.38	5.20 ± 1.50	6.00 ± 1.52^de^
	IBA	4.00 ± 1.76	6.80 ± 2.78	8.60 ± 2.54	10.00 ± 3.11	10.60 ± 3.36^b^
	NAA	0.00 ± 0.00	2.80 ± 1.88	4.60 ± 1.44	5.60 ± 1.50	6.60 ± 1.50^d^

MS012	Control	1.40 ± 0.75	1.40 ± 0.75	2.60 ± 1.03	3.80 ± 1.32	4.60 ± 1.57^f^
	IBA	10.60 ± 3.30	14.00 ± 4.09	16.40 ± 4.35	18.40 ± 4.63	20.80 ± 5.27^a^
	NAA	2.00 ± 0.95	5.20 ± 0.86	6.80 ± 1.07	7.00 ± 1.05	8.20 ± 0.97^c^

*Note*. Mean value within a column followed by the same letter shows no significant difference at *P* < 0.05 using Tukey test.

**Table 6:** Mean root length of three varieties treated with IBA and NAA from day 6 to day 14.

Variety	Plant hormone	Root length (cm)				
		Day 6	Day 8	Day 10	Day 12	Day 14
Mergong	Control	0.00 ± 0.00	0.08 ± 0.05	0.12 ± 0.08	0.15 ± 0.10	0.49 ± 0.13^e^
	IBA	0.31 ± 0.05	0.52 ± 0.07	0.85 ± 0.09	1.21 ± 0.11	1.56 ± 0.09^b^
	NAA	0.00 ± 0.00	0.00 ± 0.00	0.17 ± 0.08	0.57 ± 0.16	0.91 ± 0.18^d^

MS007	Control	0.27 ± 0.11	0.38 ± 0.05	0.67 ± 0.08	0.92 ± 0.09	1.32 ± 0.13^bc^
	IBA	0.31 ± 0.03	0.46 ± 0.02	0.79 ± 0.06	1.08 ± 0.07	1.50 ± 0.08^b^
	NAA	0.00 ± 0.00	0.43 ± 0.06	0.68 ± 0.08	1.03 ± 0.09	1.03 ± 0.08^cd^

MS012	Control	0.23 ± 0.05	0.37 ± 0.08	0.53 ± 0.07	0.79 ± 0.10	1.08 ± 0.13^cd^
	IBA	0.59 ± 0.02	0.84 ± 0.03	1.30 ± 0.03	1.74 ± 0.06	2.02 ± 0.06^a^
	NAA	0.31 ± 0.05	0.36 ± 0.03	0.62 ± 0.05	1.01 ± 0.08	1.22 ± 0.09^c^

*Note*. Mean value within a column followed by the same letter shows no significant difference at *P* < 0.05 using Tukey test.

**Table 7:** Mean leaf number of three varieties treated with IBA and NAA from day 6 to day 14.

Variety	Plant hormone	Leaf number				
		Day 6	Day 8	Day 10	Day 12	Day 14
Mergong	Control	0.80 ± 0.80	0.80 ± 0.80	0.80 ± 0.80	0.80 ± 0.80	1.60 ± 0.75^d^
	IBA	0.80 ± 0.80	1.20 ± 1.20	1.20 ± 1.20	2.00 ± 1.10	2.00 ± 1.10^c^
	NAA	0.00 ± 0.00	0.00 ± 0.00	0.00 ± 0.00	0.80 ± 0.50	0.80 ± 0.50^f^

MS007	Control	0.00 ± 0.00	0.00 ± 0.00	0.00 ± 0.00	0.80 ± 0.80	2.20 ± 1.50^bc^
	IBA	0.00 ± 0.00	0.60 ± 0.60	0.60 ± 0.60	2.40 ± 0.98	3.20 ± 1.50^a^
	NAA	0.00 ± 0.00	0.00 ± 0.00	0.00 ± 0.00	2.00 ± 0.00	2.40 ± 0.40^b^

MS012	Control	0.00 ± 0.00	0.00 ± 0.00	0.80 ± 0.80	1.40 ± 0.75	2.20 ± 0.80^bc^
	IBA	0.00 ± 0.00	0.00 ± 0.00	0.80 ± 0.80	1.20 ± 0.49	1.20 ± 0.80^e^
	NAA	0.00 ± 0.00	1.40 ± 0.75	1.40 ± 0.75	1.40 ± 0.75	1.60 ± 0.75^d^

*Note*. Mean value within a column followed by the same letter shows no significant difference at *P* < 0.05 using Tukey test.

**Table 8:** Mean square from analysis of variance for plant growth regulators on day 14.

Source	df	Root number	Root length	Leaf number
Variety	2	262.87**	2.59**	5.49^ns^
Plant growth regulator	2	298.40**	1.76**	1.16^ns^
Variety∗hormone	4	61.57^ns^	0.23^ns^	1.69^ns^
Error	36	40.44	0.30	4.71

Total	44			

*Note*. *indicates significant difference at *P* < 0.05; **indicates significant difference at *P* < 0.01; ^ns^indicates no significant difference at *P* > 0.05.

**Table 9:** Mean root number of three varieties treated with red, white, and yellow lights from day 6 to day 14 in mist-chamber propagation box.

Variety	Light	Root number				
		Day 6	Day 8	Day 10	Day 12	Day 14
Mergong	Control	0.00 ± 0.00	0.40 ± 0.40	0.40 ± 0.40	0.40 ± 0.40	1.20 ± 0.58^fg^
	Red	0.40 ± 0.25	2.00 ± 0.89	3.80 ± 1.24	5.20 ± 0.80	5.60 ± 0.60^b^
	White	0.00 ± 0.00	1.40 ± 0.87	1.40 ± 0.87	1.80 ± 1.20	2.20 ± 1.07^e^
	Yellow	0.00 ± 0.00	0.40 ± 0.40	1.00 ± 0.63	1.60 ± 0.81	2.00 ± 1.14^ef^

MS007	Control	0.80 ± 0.58	2.60 ± 1.08	4.00 ± 1.38	5.20 ± 1.50	6.00 ± 1.52^ab^
	Red	0.20 ± 0.20	3.40 ± 1.54	3.60 ± 1.57	3.80 ± 1.53	3.80 ± 1.53^d^
	White	0.20 ± 0.20	1.80 ± 0.97	2.00 ± 0.95	2.00 ± 0.95	2.00 ± 0.95^ef^
	Yellow	0.00 ± 0.00	0.00 ± 0.00	0.00 ± 0.00	0.40 ± 0.25	0.40 ± 0.25^g^

MS012	Control	1.40 ± 0.75	1.40 ± 0.75	2.60 ± 1.03	3.80 ± 1.32	4.60 ± 1.57^c^
	Red	0.00 ± 0.00	1.20 ± 0.58	1.40 ± 0.75	1.60 ± 0.81	1.60 ± 0.81^f^
	White	1.20 ± 1.20	1.80 ± 1.11	2.20 ± 1.24	2.20 ± 1.24	2.20 ± 1.24^e^
	Yellow	0.20 ± 0.20	5.40 ± 1.91	5.60 ± 1.86	6.80 ± 2.52	6.80 ± 2.52^a^

*Note*. Mean value within a column followed by the same letter shows no significant difference at *P* < 0.05 using Tukey test.

**Table 10:** Mean root length in three varieties treated with red, white, and yellow lights from day 6 to day 14 in mist-chamber propagation box.

Variety	Light	Root length (cm)				
		Day 6	Day 8	Day 10	Day 12	Day 14
Mergong	Control	0.00 ± 0.00	0.08 ± 0.05	0.12 ± 0.08	0.15 ± 0.10	0.49 ± 0.13^d^
	Red	0.10 ± 0.06	0.54 ± 0.11	0.67 ± 0.11	0.93 ± 0.11	1.14 ± 0.15^b^
	White	0.00 ± 0.00	0.29 ± 0.08	0.65 ± 0.16	0.93 ± 0.27	1.35 ± 0.35^ab^
	Yellow	0.00 ± 0.00	0.10 ± 0.07	0.20 ± 0.07	0.39 ± 0.11	0.63 ± 0.13^cd^

MS007	Control	0.27 ± 0.11	0.38 ± 0.05	0.67 ± 0.08	0.92 ± 0.09	1.32 ± 0.13^ab^
	Red	0.04 ± 0.04	0.37 ± 0.05	0.60 ± 0.07	0.82 ± 0.08	0.99 ± 0.09^bc^
	White	0.04 ± 0.04	0.41 ± 0.07	0.68 ± 0.16	0.89 ± 0.18	1.06 ± 0.21^b^
	Yellow	0.00 ± 0.00	0.00 ± 0.00	0.00 ± 0.00	0.10 ± 0.06	0.20 ± 0.13^e^

MS012	Control	0.23 ± 0.05	0.37 ± 0.08	0.53 ± 0.07	0.79 ± 0.10	1.08 ± 0.13^b^
	Red	0.00 ± 0.00	0.20 ± 0.07	0.42 ± 0.10	0.58 ± 0.12	0.72 ± 0.14^c^
	White	0.25 ± 0.07	0.65 ± 0.20	1.03 ± 0.26	1.23 ± 0.27	1.40 ± 0.27^ab^
	Yellow	0.08 ± 0.08	0.71 ± 0.09	1.26 ± 0.15	1.39 ± 0.15	1.50 ± 0.14^a^

*Note*. Mean value within a column followed by the same letter shows no significant difference at *P* < 0.05 using Tukey test.

**Table 11:** Mean leaf number of three varieties treated with red, white, and yellow lights from day 6 to day 14 in mist-chamber propagation box.

Variety	Light	Leaf number				
		Day 6	Day 8	Day 10	Day 12	Day 14
Mergong	Control	0.80 ± 0.80	0.80 ± 0.80	0.80 ± 0.80	0.80 ± 0.80	1.60 ± 0.75^e^
	Red	0.80 ± 0.80	1.20 ± 0.80	2.80 ± 1.20	4.00 ± 1.10	4.40 ± 0.75^a^
	White	0.40 ± 0.40	1.00 ± 0.63	1.40 ± 0.51	2.40 ± 0.93	2.60 ± 0.87^d^
	Yellow	0.80 ± 0.49	0.80 ± 0.49	1.20 ± 0.80	2.80 ± 0.80	3.00 ± 0.89^c^

MS007	Control	0.00 ± 0.00	0.00 ± 0.00	0.00 ± 0.00	0.80 ± 0.80	2.20 ± 1.50^d^
	Red	0.20 ± 0.20	1.60 ± 0.98	2.80 ± 1.20	2.80 ± 1.20	2.80 ± 1.20^cd^
	White	0.00 ± 0.00	0.40 ± 0.25	2.20 ± 0.66	2.20 ± 0.66	2.20 ± 0.66^d^
	Yellow	0.00 ± 0.00	0.00 ± 0.00	0.00 ± 0.00	1.40 ± 0.87	1.40 ± 0.87^ef^

MS012	Control	0.00 ± 0.00	0.00 ± 0.00	0.80 ± 0.80	1.40 ± 0.75	2.20 ± 0.80^d^
	Red	0.00 ± 0.00	0.00 ± 0.00	0.00 ± 0.00	1.80 ± 1.56	1.80 ± 1.56^de^
	White	2.00 ± 1.27	2.00 ± 1.27	2.60 ± 1.25	2.60 ± 1.25	4.00 ± 1.30^ab^
	Yellow	0.00 ± 0.00	0.00 ± 0.00	0.40 ± 0.40	0.80 ± 0.80	2.20 ± 2.20^d^

*Note*. Mean value within a column followed by the same letter shows no significant difference at *P* < 0.05 using Tukey test.

**Table 12:** Mean square from analysis of variance for light treatments on day 14.

Source	df	Root number	Root length	Leaf number
Variety	2	5.85^ns^	0.34^ns^	2.82^ns^
Light	3	9.56^ns^	0.14^ns^	3.87^ns^
Variety∗light	6	33.41*	0.80*	4.68^ns^
Error	48	8.23	0.33	7.16

Total	59			

*Note*. *indicates significant difference at *P* < 0.05.

**indicates significant difference at *P* < 0.01.

^ns^indicates no significant difference at *P* > 0.05.

**Table 13:** Mean root number of three varieties treated with 50% and 100% of shade treatments from day 6 to day 14.

Variety	Shade	Root number				
		Day 6	Day 8	Day 10	Day 12	Day 14
Mergong	0 (control)	0.00 ± 0.00	0.40 ± 0.40	0.40 ± 0.40	0.40 ± 0.40	1.20 ± 0.58^e^
	50	0.00 ± 0.00	0.00 ± 0.00	0.20 ± 0.20	0.40 ± 0.25	0.40 ± 0.25^f^
	100	0.00 ± 0.00	0.00 ± 0.00	0.00 ± 0.00	0.20 ± 0.20	0.20 ± 0.20^fg^

MS007	0 (control)	0.80 ± 0.58	2.60 ± 1.08	4.00 ± 1.38	5.20 ± 1.50	6.00 ± 1.52^bc^
	50	1.00 ± 1.00	1.00 ± 1.00	1.00 ± 1.00	1.80 ± 0.86	2.00 ± 0.78^d^
	100	0.00 ± 0.00	0.00 ± 0.00	0.00 ± 0.00	0.00 ± 0.00	0.00 ± 0.00

MS012	0 (control)	1.40 ± 0.75	1.40 ± 0.75	2.60 ± 1.03	3.80 ± 1.32	4.60 ± 1.57^c^
	50	8.40 ± 0.60	9.00 ± 0.55	9.00 ± 0.55	9.00 ± 0.55	9.00 ± 0.55^a^
	100	2.00 ± 0.84	2.60 ± 0.60	3.60 ± 0.81	4.80 ± 1.16	6.60 ± 1.03^b^

*Note*. Mean value within a column followed by the same letter shows no significant difference at *P* < 0.05 using Tukey test.

**Table 14:** Mean root length of three varieties treated with 50% and 100% of shade treatments from day 6 to day 14.

Variety	Shade	Root length (cm)				
		Day 6	Day 8	Day 10	Day 12	Day 14
Mergong	0 (control)	0.00 ± 0.00	0.08 ± 0.05	0.12 ± 0.08	0.15 ± 0.10	0.49 ± 0.13^e^
	50	0.00 ± 0.00	0.00 ± 0.00	0.16 ± 0.16	0.42 ± 0.33	0.76 ± 0.52^d^
	100	0.00 ± 0.00	0.00 ± 0.00	0.00 ± 0.00	0.10 ± 0.10	0.20 ± 0.20^f^

MS007	0 (control)	0.27 ± 0.11	0.38 ± 0.05	0.67 ± 0.08	0.92 ± 0.09	1.32 ± 0.13^bc^
	50	0.33 ± 0.12	0.61 ± 0.20	0.72 ± 0.24	1.03 ± 0.24	1.48 ± 0.21^b^
	100	0.00 ± 0.00	0.00 ± 0.00	0.00 ± 0.00	0.00 ± 0.00	0.00 ± 0.00

MS012	0 (control)	0.23 ± 0.05	0.37 ± 0.08	0.53 ± 0.07	0.79 ± 0.10	1.08 ± 0.13^c^
	50	0.87 ± 0.05	1.22 ± 0.07	1.65 ± 0.10	1.98 ± 0.12	2.23 ± 0.14^a^
	100	0.36 ± 0.07	0.54 ± 0.05	0.73 ± 0.07	0.75 ± 0.09	0.89 ± 0.11^cd^

*Note*. Mean value within a column followed by the same letter shows no significant difference at *P* < 0.05 using Tukey test.

**Table 15:** Mean leaf number of three varieties treated with 50% and 100% shade treatments from day 6 to day 14.

Variety	Shade	Leaf number				
		Day 6	Day 8	Day 10	Day 12	Day 14
Mergong	0 (control)	0.80 ± 0.80	0.80 ± 0.80	0.80 ± 0.80	0.80 ± 0.80	1.60 ± 0.75^c^
	50	0.00 ± 0.00	0.00 ± 0.00	0.80 ± 0.80	1.20 ± 1.20	1.20 ± 1.20^cd^
	100	0.20 ± 0.20	0.20 ± 0.20	0.20 ± 0.20	0.80 ± 0.80	0.80 ± 0.80^d^

MS007	0 (control)	0.00 ± 0.00	0.00 ± 0.00	0.00 ± 0.00	0.80 ± 0.80	2.20 ± 1.50^bc^
	50	0.80 ± 0.80	1.20 ± 1.20	2.00 ± 1.10	2.20 ± 1.11	2.40 ± 1.12^b^
	100	0.00 ± 0.00	0.00 ± 0.00	0.00 ± 0.00	0.00 ± 0.00	0.00 ± 0.00

MS012	0 (control)	0.00 ± 0.00	0.00 ± 0.00	0.80 ± 0.80	1.40 ± 0.75	2.20 ± 0.80^bc^
	50	1.60 ± 0.40	2.40 ± 0.40	3.00 ± 0.45	3.00 ± 0.45	3.00 ± 0.45^a^
	100	0.00 ± 0.00	0.00 ± 0.00	0.00 ± 0.00	0.00 ± 0.00	0.00 ± 0.00

*Note*. Mean value within a column followed by the same letter shows no significant difference at *P* < 0.05 using Tukey test.

**Table 16:** Mean square from analysis of variance on day 14 for shade treatment.

Source	df	Root number	Root length	Leaf number
Variety	2	146.07**	3.20**	1.09^ns^
Shade	2	12.87^ns^	3.77**	16.96*
Variety∗shade	4	29.73**	0.79^ns^	2.39^ns^
Error	36	3.98	0.31	3.88

Total	44			

*Note*. *indicates significant difference at *P* < 0.05.

**indicates significant difference at *P* < 0.01.

^ns^indicates no significant difference at *P* > 0.05.
